# The cilia-regulated proteasome and its role in the development of ciliopathies and cancer

**DOI:** 10.1186/s13630-016-0035-3

**Published:** 2016-06-10

**Authors:** Christoph Gerhardt, Tristan Leu, Johanna Maria Lier, Ulrich Rüther

**Affiliations:** Institute for Animal Developmental and Molecular Biology, Heinrich-Heine University Düsseldorf, 40225 Düsseldorf, Germany

**Keywords:** Cilia, Proteasome, Tumor, RPGRIP1L, SHH, PDGFRα, NOTCH, TGFβ, WNT, Signaling

## Abstract

The primary cilium is an essential structure for the mediation of numerous signaling pathways involved in the coordination and regulation of cellular processes essential for the development and maintenance of health. Consequently, ciliary dysfunction results in severe human diseases called ciliopathies. Since many of the cilia-mediated signaling pathways are oncogenic pathways, cilia are linked to cancer. Recent studies demonstrate the existence of a cilia-regulated proteasome and that this proteasome is involved in cancer development via the progression of oncogenic, cilia-mediated signaling. This review article investigates the association between primary cilia and cancer with particular emphasis on the role of the cilia-regulated proteasome.

## Background

The precise coordination and regulation of cellular processes is the basis for the development and the homeostasis of a multi-cellular organism. To ensure this high precision, the cell makes use of a special structure that is observed as a 1–10-μm-long cellular evagination—the primary cilium. Simplified, the structure of the cilium consists of three different compartments—the basal body (BB), the axoneme, and the transition zone (TZ). The BB is a remodeled mother centriole from which the ciliary scaffold (axoneme) consisting of circularly arranged nine doublet microtubules arises. The intermediate region from the BB to the axoneme is a short area of 0.5 μm called TZ. The primary cilium plays a decisive role in the initiation of the molecular mechanisms underlying cellular processes like proliferation, apoptosis, migration, differentiation, transcription, and the determination of cell polarity [1, 2]. Consequently, ciliary dysfunction results in severe diseases collectively summarized as ciliopathies. Well-known ciliopathies are: Joubert syndrome (JBTS), Leber’s congenital amaurosis (LCA), Senior–Løken syndrome (SLS), nephronophthisis (NPHP), Meckel–Gruber syndrome (MKS), Bardet–Biedl syndrome (BBS), orofaciodigital syndrome type 1 (OFD1), Alström syndrome (ALS), Jeune asphyxiating thoracic dystrophy (JATD), Ellis–van Creveld syndrome (EVC), and sensenbrenner syndrome (cranioectodermal dysplasia [CED]) [3]. Additionally, cilia are linked to cancer. The current, general view is that, on the one hand, primary cilia mediate oncogenic signaling and, on the other hand, cilia are lost in some types of cancer. In this review article, the role of cilia in cancer development will be discussed with particular regard to the cilia-controlled proteasome. The focus is on the question: What is the significance of the cilia-regulated proteasome in terms of cancerogenesis?

## Primary cilia, intercellular signaling, and cancer

**Fig. 1:**
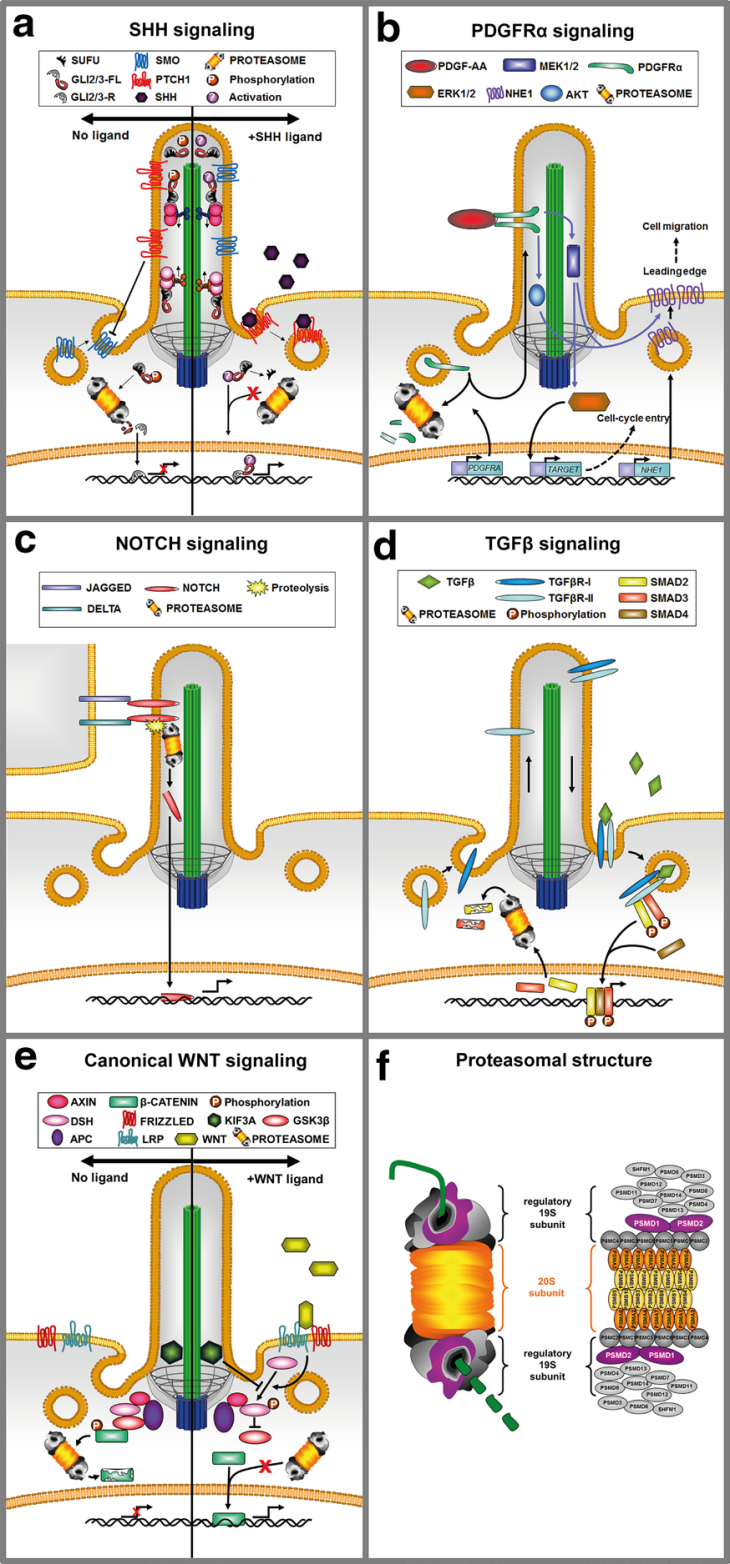
Cilia-mediated signaling pathways whose proper regulation is dependent on the proteasome and the structure of the proteasome. **a**–**e** SHH, PDGFRα, NOTCH, TGFβ, and canonical WNT signaling is transduced by primary cilia. **a** In the absence of the ligand SHH, SMO remains in cytoplasmic vesicles and is inhibited by PTCH1. As a result, GLI2 and GLI3 (forming a complex with SUFU) are phosphorylated most likely within the cilium and subsequently get proteolytically processed to their repressor forms (GLI2/3-R) by the proteasome at the ciliary base. In turn, GLI2/3-R translocate into the nucleus and represses the expression of SHH target genes. Importantly, GLI3 is the predominant repressor. When SHH binds to its receptor PTCH1, the SHH/PTCH1 complex leaves the cilium and PTCH1 is not able to inhibit the action of SMO any longer. Thereupon, SMO is transported into the cilium and converts the full-length forms of GLI2 and GLI3 (GLI2/3-FL) into their activator forms. In the course of this conversion process, SUFU dissociates from the complex enabling the GLI2 and GLI3 activator forms to induce SHH target gene expression. **b** In the ciliary membrane, PDGFRα is bound by its ligand PDGF-AA and subsequently becomes dimerized and phosphorylated. The phosphorylation of PDGFRα induces the activation of the MEK 1/2-ERK 1/2 and AKT/PKB signaling pathways. **c** Initiating NOTCH signaling, the extracellular domain of a NOTCH ligand (JAGGED or DELTA) binds to the NOTCH receptor which is located in the ciliary membrane. As a result, the NOTCH receptor undergoes a three-step cleavage and finally releases the NOTCH intracellular domain (NIC). NIC enters the nucleus and activates NOTCH target genes. **d** The receptors of the TGFβ pathway, TGFβ-RI and TGFβ-RII, are located at the ciliary base. When the TGFβ ligand binds to the receptors a heterotetrameric receptor complex composed of TGFβ-RI and TGFβ-RII is formed and activated. This activation results in the phosphorylation and activation of SMAD2 and SMAD3. The phosphorylated SMADs 2 and 3 associate with a co-SMAD called SMAD4. Afterwards, the complex consisting of SMAD2, 3, and 4 enters the nucleus and activates TGFβ target genes. **e** In the inactive state of the canonical WNT pathway, a destruction complex consisting of APC and AXIN triggers the phosphorylation of β-catenin by GSK3. After this phosphorylation event, β-catenin gets ubiquitinated and finally degraded. In the active state, WNT ligands bind to FRIZZLED and LRP receptors leading to the activation of DSH. DSH recruits the destruction complex to the plasma membrane, thereby interfering phosphorylation of β-catenin. Afterwards, β-catenin translocates into the nucleus and activates canonical WNT target gene expression. Primary cilia restrict canonical WNT signaling because the ciliary protein KIF3A is able to inhibit the phosphorylation of DSH. **f** The proteasome consists of the catalytic 20S subunit and two regulatory 19S subunits. The 20S subunit displays a cylindrical arrangement of four stacked heptameric rings. Each ring is composed of seven α and β subunits, respectively. Only three subunits (PSMB8-10) display a proteolytic activity equipping the proteasome with trypsin-like, chymotrypsin-like, and caspase-like abilities. The 19S subunit can be subdivided into two subcomplexes: a base complex (being constituted of six ATPases [PSMC1-6] and three non-ATPases [PSMD1, 2 and 4]) and a lid complex (consisting of nine non-ATPases [PSMD3, 6-8, 11-14, and SHFM1])

Primary cilia mediate intercellular signaling pathways which are involved in the regulation of cellular processes and the formation and maintenance of all organs and structures within the human body. Cancer is characterized by uncontrolled cell division as well as an impaired ability to undergo apoptosis [4] and because it develops as a result of altered intra- and intercellular signaling, disturbances of cilia-mediated signaling pathways can result in tumor formation [5–7]. While it seems as if canonical WNT signaling is restricted by cilia [8–10], various publications have shown cilia-dependent mediation of sonic hedgehog (SHH), platelet-derived growth factor receptor-α (PDGFRα), NOTCH, transforming growth factor (TGF)-β, and non-canonical WNT signaling (Fig. 1a–e) [8, 11–18].

Of all the investigated associations between primary cilia and signaling pathways, the relationship between primary cilia and SHH signaling is the best studied. In SHH signaling, the 12-pass transmembrane protein patched1 (PTCH1) is located in the ciliary membrane of vertebrates (Fig. 1a). When the SHH ligand binds to its receptor PTCH1, the SHH/PTCH1 complex leaves the cilium. As a consequence, the seven-transmembrane protein smoothened (SMO) is allowed to accumulate in the ciliary membrane and to invoke glioblastoma (GLI) transcription factors. Three GLI isoforms exist in vertebrates—GLI1, 2, and 3. The GLI proteins regulate the expression of SHH target genes and thereby cell proliferation, differentiation, survival and growth [19, 20]. While GLI1 exclusively functions as a constitutive transcriptional activator [21, 22], GLI2 and GLI3 can serve as an activator or a repressor [23]. In the presence of SHH, full-length GLI2 (GLI2-185) and GLI3 (GLI3-190) proteins are converted into a transcriptional activator (GLI2-A and GLI3-A, respectively) most likely by modifications [24, 25]. In the absence of SHH, the full-length proteins can be proteolytically processed into transcriptional repressors (GLI2-R, also known as GLI2-78, and GLI3-R, also known as GLI3-83, respectively) [26]. It was reported that GLI3-R is the predominant repressor of SHH target gene transcription [26]. The ratio of activator and repressor forms regulates cellular processes dependent on SHH signaling.

Similar to SHH signaling, activated PDGF receptors control cellular processes like proliferation, anti-apoptosis, migration, differentiation, actin reorganization, and cell growth [27–29]. The receptor PDGFRα localizes to cilia and undergoes dimerization and phosphorylation after being bound by its ligand PDGF-AA [14] (Fig. 1b). Stimulation of PDGFRα provokes the activation of signal transduction through the MEK 1/2-ERK 1/2 and AKT/PKB pathways. In the absence of cilia, PDGFRα signaling is inhibited [14]. Additionally, PDGFRα signaling is restricted by the mammalian target of rapamycin (mTOR) signaling pathway [30–32], which is also associated with cilia-mediated signaling. LKB1, a negative regulator of mTOR, localizes to cilia and its action leads to an accumulation of phosphorylated AMPK at the basal body [33]. In turn, the phosphorylation of AMPK results in the inhibition of mTOR signaling via a mechanism that is only poorly understood. Interestingly, deregulation of mTOR signaling has been described in many cancer types [34–36]. Previously, it has been demonstrated that NOTCH signaling depends on primary cilia [16, 17] (Fig. 1c). NOTCH signaling starts when the extracellular domain of a NOTCH ligand, e.g., delta-like1–4 or jagged1–2, binds to the NOTCH receptor (NOTCH1–4) [37]. A ciliary localization was shown for NOTCH1 and NOTCH3 [16, 17]. After the binding event, the NOTCH receptor undergoes a three-step cleavage and finally releases the NOTCH intracellular domain (NIC). Following this, NIC enters the nucleus and interacts with its DNA-binding cofactor RBP-J/CBF1/CSL thereby activating NOTCH target genes. NOTCH signaling controls among other proliferation and differentiation [38].

Moreover, TGFβ signaling relates to cilia [18] (Fig. 1d). Both receptors of the pathway, TGFβ-RI and TGFβ-RII, are located at the base of primary cilia. The ligand-induced formation and activation of a heterotetrameric receptor complex composed of TGFβ-RI and TGFβ-RII results in the phosphorylation and activation of the SMAD2 and SMAD3 proteins which are present at the ciliary base [18]. The phosphorylated SMADs 2 and 3 associate with a co-SMAD called SMAD4 that is also detectable at the base of cilia. Subsequently, the complex consisting of SMAD2, 3, and 4 enters the nucleus and activates TGFβ target genes. TGFβ target genes control cellular processes like proliferation, differentiation, morphogenesis, tissue homeostasis, and regeneration [39].

Primary cilia are also connected to WNT signaling [40], which can be classified as canonical (β-catenin dependent) or non-canonical (β-catenin independent). In the inactive state of the canonical WNT pathway, a destruction complex consisting of adenomatous polyposis coli (APC) and AXIN triggers the phosphorylation of β-catenin by casein kinase 1 (CK1) and glycogen synthase kinase 3 (GSK3) (Fig. 1e). Afterwards, β-catenin gets phosphorylated, ubiquitinated, and finally degraded [41]. The WNT/β-catenin pathway becomes initiated by binding of WNT ligands to frizzled (FZ) receptors and low density lipoprotein-related proteins 5/6 (LRP 5/6) and leads to the activation of the cytoplasmatic phosphoprotein disheveled (DSH). Subsequently, DSH recruits the destruction complex to the plasma membrane, thereby inhibiting phosphorylation of β-catenin. This operation of DSH enables β-catenin to translocate into the nucleus for activating target gene transcription. Several processes are controlled by canonical WNT signaling: cell fate determination, migration, proliferation, tumor suppression, and self-renewal of stem and progenitor cells [42, 43].

In contrast to canonical WNT signaling, the non-canonical WNT pathway is less well understood. Hence, it is unknown, if β-catenin-independent WNT pathways function as different distinct pathways or if these pathways form a large signaling network [44]. Like the canonical WNT pathway, it starts with a WNT ligand binding to the FZ receptor, but does not require the presence of LRP co-receptors or β-catenin. Non-canonical WNT signals are mediated through intracellular Ca^2+^ levels and involvement of RHO A, ROCK, and JNK kinase. These factors play an important role in the regulation and remodeling of the cytoskeleton and are greatly involved in the control of planar cell polarity (PCP). PCP is established by intercellular communication that regulates the composition of cells polarizing structures within the plane of a tissue, i.e., stereocilia bundle orientation in the inner ear [45]. In addition to managing cytoskeleton organization, non-canonical WNT signals regulate proliferation and migration [46].

The restriction of canonical WNT signals by cilia is likely, since DSH is constitutively phosphorylated in *Kif3a*-negative mice which are unable to assemble cilia [47]. However, non-canonical WNT signaling seems to be mediated by primary cilia [8–10]. One core PCP gene product, van gogh-like 2 (VANGL2), was found in cilia [48]. The ciliary presence of VANGL2 [48] and the finding that VANGL2 is essential for the transduction of WNT5a-induced signals to establish PCP [49] suggest that non-canonical WNT signaling might be mediated by cilia. This hypothesis is supported by data showing that disruption of BBS protein function leads to ciliary dysfunction along with perturbation of PCP [48] and that ciliopathy genes interact genetically with VANGL2 [48, 50]. In summary, these data suggest that primary cilia mediate non-canonical WNT signals and limit canonical WNT signaling [51].

Dysregulation of any of these pathways could lead to oncogenesis. In many cases, upregulation of their target gene expressions led to an increased cell proliferation, which in turn caused tumorigenesis [52–56]. One of the best studied oncogenic signaling pathways is the SHH pathway which was already analyzed in combination with cilia in cancer cells [57, 58]. In 2009, Han et al. and Wong et al. [59, 60] described the role of primary cilia in the development of medulloblastomas and basal cell carcinomas. In regard to SHH signaling, both groups showed that the absence of cilia can protect against tumorigenesis and, in addition, that the presence of cilia can be necessary for the induction of tumors. First, they induced tumorigenesis via a cell type-specific expression of an activated SMO protein. Then, they performed the experiments in mice that were unable to form cilia in the particular cell type for the formation of either medulloblastomas or basal cell carcinomas. In both cases, ciliary deficiency protected against SMO-induced tumorigenesis [59, 60].

Second, the same groups investigated the consequences of constitutively active GLI2 on tumorigenesis [59, 60]. In case of basal cell carcinoma development, constitutively active GLI2 was sufficient to induce carcinogenesis [60], while, in case of medulloblastoma development, constitutively active GLI2 did not give rise to carcinogenesis [59]. Importantly, the combination of constitutively active GLI2 and loss of cilia led to the formation of medulloblastomas [59] giving circumstantial evidence that the additional decreased amount of GLI3-R caused by ciliary absence might be necessary to induce oncogenesis. Accordingly, the activation of SHH target gene expression alone is not strong enough for driving the development of some cancer types, but in combination with an inhibited repression of SHH target gene expression by reducing the amount of GLI3-R, activation of SHH target gene expression is sufficient to induce oncogenesis. Possibly, the reason for these differences is that the importance of GLI3-R is different in diverse cancer types. Perhaps, it is even the case that the efficiency of GLI3 processing is different in different cancer types and the amount of GLI3-R varies. A decisive factor for the proteolytic processing of GLI3 is the proteasome.

## The proteasome and cancer

The proteasome functions as the catalytic component of the ubiquitin–proteasome system and consists of 19S and 20S subunits (Fig. 1f). Proteins destined to get degraded or proteolytically processed become phosphorylated and ubiquitinated. Polyubiquitin conjugation is realized by a cooperation of an ubiquitin-activating enzyme (E1), an ubiquitin conjugation enzyme (E2), and an ubiquitin ligase (E3). In search of molecular mechanisms underlying carcinogenesis, it was reported that while E1 was never found to be associated with tumor formation, deregulation of E2 and especially E3 was detected in tumors [61]. In some cases, E3 ligases are inactivated leading to a stabilization of oncogene products. In other cases, E3 ligases are overexpressed causing an increased degradation of tumor suppressor proteins [62]. Finally, ubiquitinated proteins bind to the 19S regulatory complex. Hereafter, they are degraded by the multiple peptidase activities containing 20S subunit [63]. Besides the degradation of proteins, the proteasome is able to proteolytically process proteins. A well-studied processing event is the transformation of full-length GLI3 into its shorter repressor form. This process depends on a three-part signal [64]. The first processing signal is the zinc finger domain of the GLI3 protein, which serves as a physical barrier to the proteasome. It prevents degradation of the GLI3 protein and is an essential prerequisite for GLI3 processing. Accordingly, the proteasome is not the factor which distinguishes degradation from processing, but the protein which is degraded or processed determines its fate via its sequence. The linker sequence which expands between the zinc finger domain and the lysines of the degron sequence functions as the second processing signal. Most likely, the proteasome binds to the linker area, which is assumed to be a proteasome initiation region. The degron is the third processing signal and the starting point of proteasomal processing.

In addition to its role in SHH signaling, the proteasome is important for the proper course of several cilia-mediated signaling pathways. It was reported that PDGFRα signaling is upregulated in cancer cells due to an elevated amount of PDGFRα [65]. In these cells, HSP90 and the co-chaperone CDC37 form a complex with PDGFRα, making it inaccessible to proteasomal degradation (Fig. 1b). Previously, it was reported that the amount of PDGFRα could also be decreased in kidney tumors, while the amount of mTOR is increased and mTOR signaling is upregulated [30, 31, 66]. Because mTOR regulates PDGFRα signaling negatively by reducing the amount of PDGFRα [30] and mTOR governs proteasomal activity positively [67], it is conceivable that mTOR controls the PDGFRα amount via regulating proteasomal activity. If this hypothesis is true, it could be possible that cancer with a high PDGFRα amount is characterized by downregulated mTOR signaling. As far as we know, the evidence for this possibility has not been found yet. The proteasome is also involved in the regulation of NOTCH signaling, because it controls the NIC amount [68, 69] (Fig. 1c). In lung adenocarcinoma cells, proteasomal degradation of NIC is impaired resulting in enhanced cell proliferation and hence tumorigenesis [70]. Furthermore, TGFβ signaling requires the services of the proteasome. Phosphorylated SMAD2 and SMAD3, the central transducers of the pathway, are inactivated by proteasomal degradation [71, 72] (Fig. 1d). Accordingly, reduced proteasomal degradation of these SMADs gives rise to hyperproliferative diseases like cancer [71]. As previously mentioned, canonical WNT signaling is most likely restricted by primary cilia [47]. At the base of these cilia, the proteasome degrades β-catenin that is phosphorylated at Ser33, Ser37, and Thr41 [47, 50] (Fig. 1e). In some tumors, this kind of phosphorylation is prevented by mutations resulting in a stabilization of β-catenin which then is able to activate the transcription of many oncogenes [73, 74]. Consequently, canonical WNT signaling is not only restricted by primary cilia but also by proteasomal degradation of β-catenin. As opposed to the just described signaling pathways, an essential role of the proteasome in non-canonical WNT signaling has never been described.

In sum, a decreased proteasomal activity causes a deregulation of signaling pathways, leading to an increased cell proliferation resulting in the development of cancer. However, numerous studies show that proteasomal activity is enhanced in cancer cells [75–89] representing an obvious discrepancy. A plethora of point mutations in cancer genomes lead to a very high number of misfolded proteins [90]. It was hypothesized that the cell faces this enormous boost of useless and even harmful proteins with enhanced proteasome-mediated degradation [91]. Moreover, estimates suggest that 90 % of human solid tumors comprise cells with more than two copies of one or more chromosomes [92]. For this reason, a huge surplus of proteins is produced in these cells resulting in a cellular protein imbalance [93, 94]. Consequently, many proteins are not able to form a stable conformation and get degraded by the proteasome [95, 96]. Thus, cancer cells show an increased proteasomal activity due to various reasons. This phenomenon has been designated as “proteotoxic crisis” [91]. Based on this knowledge, proteasome inhibitors are used in anti-cancer therapies [97].

However, there is a unique class of cancer cells with a decreased proteasomal activity in which the use of proteasome inhibitors would be counterproductive. Reduced proteasomal activity is a hallmark of several cancer stem cells (CSCs) [98–103]. In contrast, glioma stem-like cells (GSCs) show an increase of proteasomal activity [104] suggesting that proteasomal activity may vary among types of CSCs. But it is doubtful whether GSCs belong to the group of CSCs because they maintain only some properties of CSCs [105]. CSCs (also known as cancer-initiating cells) are part of a new understanding in terms of tumorigenesis. In contrast to the “stochastic model” in which every cancer cell of a tumor is capable of repopulating the entire tumor because of its property of self-renewal, this model conveys the idea that only a small group of cancer cells (CSCs) within a tumor has the ability to repopulate the tumor and that the progeny of these cells loses this ability [106–109]. Even in the course of chemotherapy, CSCs are able to survive and initiate the re-growth of tumors [110, 111]. Thus, CSCs are the reason for the resistance of tumors to conventional anti-cancer therapies. Consequently, it is a challenging task for the current research to develop new anti-cancer therapies which target CSCs [111]. In the development of this type of anti-cancer therapies, a broad spectrum of pharmaceutical compounds were tested. Interestingly, natural dietary compounds came into focus [112]. Since proteasomal activity is reduced in most CSCs and since the decisive signals thought to underlie the self-renewal mechanism of the CSCs are, inter alia, SHH signaling, PDGFRα signaling, NOTCH signaling, TGFβ signaling, and WNT signaling [106, 113–119], one of these compounds is sulforaphane (SFN; 1-isothiocyanato-4(R)-methylsulfinylbutane), an ingredient of broccoli, which functions as a proteasome activator [120]. In 2010, Li et al. [101] tested the effect of SFN on breast cancer cells. They came up with the conclusion that the SFN treatment downregulated canonical WNT signaling by promoting proteasomal degradation of β-catenin in CSCs. The SFN treatment eliminated breast CSCs [101], indicating that the decreased proteasomal activity is essential for the survival of CSCs and that SFN could be an effective drug in anti-cancer stem cell therapies.

## Primary cilia and the proteasome

After reviewing the connections between primary cilia and cancer, as well as the proteasome and cancer, the relationship between primary cilia and the proteasome should be investigated in order to determine the molecular mechanisms underlying cancer development. As early as 2003, it was suggested that although proteasomes exist almost ubiquitously within the cytoplasm and the nucleus, “their function is likely to be different at different cellular locations” and that “this probably depends on post-translational modifications of proteasomal subunits and on their association and interaction with specific regulatory proteins” [121]. In 2007, Gerdes et al. [50] reported that the ciliary protein BBS4 is involved in the proteasomal degradation of cytoplasmic β-catenin, the mediator of canonical WNT signaling. In the following years, interactions of a whole range of ciliary proteins with proteasomal components were identified (Table 1) indicating a possible link between cilia and the proteasome. In this context, it was shown that the ciliary proteins BBS1, BBS2, BBS4, BBS6, BBS7, BBS8, and OFD1 interact directly with different proteasomal components [122]. The loss of BBS4, BBS7, and OFD1 leads to a reduced proteasomal activity, respectively, impairing intercellular signaling pathways [50, 122, 123]. In search of the molecular reason for the depleted proteasomal activity, Liu et al. [122] measured a decreased amount of different proteasomal components in the absence of BBS4 and OFD1, respectively, demonstrating that these proteins control the composition of the proteasome. Since all these proteins localize to the basal body which is equivalent to the mother centriole in ciliary absence, the authors of this study refer to the effect of these proteins on the “centrosomal proteasome” [122]. The existence of a centrosome-associated proteasome was already shown before [124, 125]. Thus, the question arises whether the cilium is important for proteasomal function or whether it rests on the centrosome alone to regulate proteasomal activity. Three components of the 19S proteasomal subunit (PSMD2, PSMD3, and PSMD4) were detected at the BB of mouse embryonic fibroblast (MEF) cilia [126]. However, the detection of proteasomal components at the BB is not sufficient to answer this question; it might be that the centrosomal and the putative ciliary proteasome (a proteasome that functions cilia dependent) are one and the same. Remarkably, a component of the 20S proteasomal subunit (PSMA5) was found along the whole cilium increasing the likelihood of a ciliary involvement in proteasome assembly or function [126]. Interestingly, the ubiquitin conjugation system has been described in flagella of the single-cell green alga *Chlamydomonas reinhardtii* but, in contrast to the cilia of MEFs, no proteasomal components were detected in these flagella [127] indicating that the potential ciliary proteasome developed later in evolution and might even be vertebrate specific. Using the G-LAP-Flp purification strategy in mammalian cell lines [128] which ensures high-confidence proteomics, numerous interactions of the transition zone proteins INVS (also known as NPHP2), IQCB1 (also known as NPHP5), and RPGRIP1L (also known as FTM, NPHP8, or MKS5) with different components of the proteasome were detected [129]. It was already shown that these three proteins are located at the centrosomes during mitosis [126, 129–132] enabling a putative interaction with a component of the centrosomal proteasome. In *Rpgrip1l*-negative MEFs and limbs of mouse embryos, a reduced proteasomal activity was quantified at the ciliary base. In contrary to the situation in the absence of BBS4 and OFD1 which was characterized by a reduced overall cellular proteasomal activity, RPGRIP1L deficiency results in a decreased proteasomal activity exclusively at the base of cilia (in ciliary absence, the proteasomal activity at centrosomes of *Rpgrip1l*^−/−^ MEFs is unaltered) demonstrating the existence of a ciliary proteasome [122, 126]. This study could draw the attention from the connection between centrosome and proteasome to the link between primary cilia and proteasome. Contrary to the situation in the absence of BBS4 and OFD1 which was characterized by a depletion of proteasomal components, RPGRIP1L deficiency results in an accumulation of proteasomal 19S and 20S subunit components at the ciliary base [122, 126]. Another difference between these ciliary proteins is the choice of their proteasomal interaction partners. While RPGRIP1L and OFD1 have been shown to interact with components of the 19S proteasomal subunit, BBS4 interacts with components of the 19S as well as 20S proteasomal subunits (Table 1). All these findings indicate that ciliary proteins use different mechanisms with which they regulate proteasomal activity.

**Table 1:** Interactions between ciliary proteins and proteasomal components

Ciliary protein (localization)	Proteasomal component (subunit)	Cell type	Source
BBS1 (basal body)	PSMB1 (20S subunit)	C57BL/6 testis	[122]
BBS1 (basal body)	RPN10 (19S subunit)	C57BL/6 testis	[122]
BBS1 (basal body)	RPN13 (19S subunit)	C57BL/6 testis	[122]
BBS1 (basal body)	RPT6 (19S subunit)	C57BL/6 testis	[122]
BBS1 (basal body)	PA28 gamma (19S subunit)	C57BL/6 testis	[122]
BBS2 (basal body)	PSMB1 (20S subunit)	C57BL/6 testis	[122]
BBS2 (basal body)	RPN10 (19S subunit)	C57BL/6 testis	[122]
BBS2 (basal body)	RPN13 (19S subunit)	C57BL/6 testis	[122]
BBS2 (basal body)	RPT6 (19S subunit)	C57BL/6 testis	[122]
BBS2 (basal body)	PA28 gamma (19S subunit)	C57BL/6 testis	[122]
BBS4 (basal body)	PSMB1 (20S subunit)	C57BL/6 testis	[122]
BBS4 (basal body)	RPN10 (19S subunit)	C57BL/6 testis	[122]
BBS4 (basal body)	RPN13 (19S subunit)	C57BL/6 testis	[122]
BBS4 (basal body)	RPT6 (19S subunit)	C57BL/6 testis	[122]
BBS4 (basal body)	PA28 gamma (19S subunit)	C57BL/6 testis	[122]
BBS6 (basal body)	PSMB1 (20S subunit)	C57BL/6 testis	[122]
BBS6 (basal body)	RPN10 (19S subunit)	C57BL/6 testis	[122]
BBS6 (basal body)	RPN13 (19S subunit)	C57BL/6 testis	[122]
BBS6 (basal body)	RPT6 (19S subunit)	C57BL/6 testis	[122]
BBS6 (basal body)	PA28 gamma (19S subunit)	C57BL/6 testis	[122]
BBS7 (basal body)	PSMB1 (20S subunit)	C57BL/6 testis	[122]
BBS7 (basal body)	RPN10 (19S subunit)	C57BL/6 testis	[122]
BBS7 (basal body)	RPN13 (19S subunit)	C57BL/6 testis	[122]
BBS7 (basal body)	RPT6 (19S subunit)	C57BL/6 testis	[122]
BBS7 (basal body)	PA28 gamma (19S subunit)	C57BL/6 testis	[122]
BBS8 (basal body)	PSMB1 (20S subunit)	C57BL/6 testis	[122]
BBS8 (basal body)	RPN10 (19S subunit)	C57BL/6 testis	[122]
BBS8 (basal body)	RPN13 (19S subunit)	C57BL/6 testis	[122]
BBS8 (basal body)	RPT6 (19S subunit)	C57BL/6 testis	[122]
BBS8 (basal body)	PA28 gamma (19S subunit)	C57BL/6 testis	[122]
INVS (transition zone + inversin compartment)	PSMD9 (19S subunit)	IMCD3	[129]
IQCB1 (transition zone + basal body)	PSMB1 (20S subunit)	3T3	[129]
IQCB1 (transition zone + basal body)	PSMA3 (20S subunit)	3T3	[129]
IQCB1 (transition zone + basal body)	PSMB6 (20S subunit)	3T3	[129]
IQCB1 (transition zone + basal body)	PSMB5 (20S subunit)	3T3	[129]
IQCB1 (transition zone + basal body)	PSMA6 (20S subunit)	3T3	[129]
IQCB1 (transition zone + basal body)	PSMB7 (20S subunit)	IMCD3	[129]
IQCB1 (transition zone + basal body)	PSMA5 (20S subunit)	IMCD3	[129]
IQCB1 (transition zone + basal body)	PSMB6 (20S subunit)	IMCD3	[129]
IQCB1 (transition zone + basal body)	PSMA4 (20S subunit)	IMCD3	[129]
IQCB1 (transition zone + basal body)	PSMB2 (20S subunit)	IMCD3	[129]
IQCB1 (transition zone + basal body)	PSMB5 (20S subunit)	IMCD3	[129]
IQCB1 (transition zone + basal body)	PSMA7 (20S subunit)	IMCD3	[129]
IQCB1 (transition zone + basal body)	PSMA1 (20S subunit)	IMCD3	[129]
IQCB1 (transition zone + basal body)	PSMB3 (20S subunit)	IMCD3	[129]
IQCB1 (transition zone + basal body)	PSMB1 (20S subunit)	IMCD3	[129]
IQCB1 (transition zone + basal body)	PSME4 (proteasome activator protein)	IMCD3	[129]
IQCB1 (transition zone + basal body)	PSMA3 (20S subunit)	IMCD3	[129]
IQCB1 (transition zone + basal body)	PSMA7 (20S subunit)	IMCD3	[129]
IQCB1 (transition zone + basal body)	PSMB4 (20S subunit)	IMCD3	[129]
IQCB1 (transition zone + basal body)	PSMA2 (20S subunit)	IMCD3	[129]
IQCB1 (transition zone + basal body)	PSMA6 (20S subunit)	IMCD3	[129]
IQCB1 (transition zone + basal body)	PSMB4 (20S subunit)	RPE	[129]
IQCB1 (transition zone + basal body)	PSMA1 (20S subunit)	RPE	[129]
IQCB1 (transition zone + basal body)	PSMA2 (20S subunit)	RPE	[129]
IQCB1 (transition zone + basal body)	PSMA3 (20S subunit)	RPE	[129]
IQCB1 (transition zone + basal body)	PSMA4 (20S subunit)	RPE	[129]
IQCB1 (transition zone + basal body)	PSMA5 (20S subunit)	RPE	[129]
IQCB1 (transition zone + basal body)	PSMA6 (20S subunit)	RPE	[129]
IQCB1 (transition zone + basal body)	PSMA7 (20S subunit)	RPE	[129]
IQCB1 (transition zone + basal body)	PSMB1 (20S subunit)	RPE	[129]
IQCB1 (transition zone + basal body)	PSMB2 (20S subunit)	RPE	[129]
IQCB1 (transition zone + basal body)	PSMB3 (20S subunit)	RPE	[129]
IQCB1 (transition zone + basal body)	PSMB5 (20S subunit)	RPE	[129]
IQCB1 (transition zone + basal body)	PSMB6 (20S subunit)	RPE	[129]
IQCB1 (transition zone + basal body)	PSMB7 (20S subunit)	RPE	[129]
OFD1 (basal body)	RPT6 (19S subunit)	C57BL/6 testis	[122]
RPGRIP1L (transition zone)	PSMC2 (19S subunit)	IMCD3	[129]
RPGRIP1L (transition zone)	PSMC5 (19S subunit)	IMCD3	[129]
RPGRIP1L (transition zone)	PSMD11 (19S subunit)	IMCD3	[129]
RPGRIP1L (transition zone)	PSMD3 (19S subunit)	IMCD3	[129]
RPGRIP1L (transition zone)	PSMD2 (19S subunit)	HEK293T	[126]
RPGRIP1L (transition zone)	PSMD2 (19S subunit)	NIH/3T3	[126]

Mutations in *RPGRIP1L*, *BBS4*, and *OFD1* give rise to very severe ciliopathies which often lead to death in men and mice [133–143]. These ciliary proteins regulate proteasomal activity [50, 122, 126] and the proteasome is involved in the development and function of numerous organs and structures of the human body [144–146]. Therefore, reduced activity of the cilia-regulated proteasome is a potential cause of ciliopathies. Appropriately, in silico studies using a systematic network-based approach to work out the “cilia/centrosome complex interactome (CCCI)” revealed that the greatest community of the CCCI is composed of proteasomal components [147]. Thus, it is likely that the relationship between ciliary proteins and the proteasome is of great importance. Further evidence for this importance is given by rescue experiments in vivo. The injection of proteasomal component mRNA or SFN treatment restored defective convergent extension and somatic definition in zebrafish embryos treated with *bbs4* or *ofd1* morpholinos [122]. Additionally, it could be shown that the introduction of a constitutively active Gli3-R protein (Gli3^Δ699^) rescues telencephalic patterning, olfactory bulb morphogenesis, and the agenesis of the corpus callosum in *Rpgrip1l*-negative mouse embryos [148, 149]. Together, these data demonstrate that a decreased activity of the cilia-regulated proteasome is responsible for the development of ciliopathies in these model organisms. Future studies should address if this is also true for human ciliopathies.

## Does the cilia-regulated proteasome play a role in the development of cancer?

Several studies have focused on the association between cancer and ciliary presence [150–160]. Since a reduced number of cilia was detected in different cancer types [57–60, 150–156, 158, 159, 161], it was reported that tumorigenesis results in a reduced cilia frequency in some cancer types. Until now, it is unknown why some cancer cell types possess cilia and others not (Table 2). Although the absence of cilia is able to correct effects of an oncogenic initiating event that lies upstream of ciliary action [59, 60], the loss of cilia is not the only solution to treat cancerogenesis. If the oncogenic initiating event lies downstream of ciliary action, therapeutic targeting of cilia would not help in the development of cancer therapies. Accordingly, genetic screening for the oncogenic initiator might be the most important point to design effective anti-cancer therapies. In this context, it would be an interesting question for future investigations whether ciliary genes are mutated in patients suffering from cancer. It was previously reported that the ciliary gene *RPGRIP1L* might serve as a tumor suppressor gene because *RPGRIP1L* was downregulated in human hepatocellular carcinoma [162]. Mechanistically, RPGRIP1L is thought to suppress tumor cell transformation in part by regulating MAD2, a mitotic checkpoint protein whose inactivation is realized by the proteasome [162, 163]. Since knockdown of *RPGRIP1L* led to an increased amount of MAD2, the function of RPGRIP1L as a controller of ciliary proteasome activity could be of great importance in the prevention of human hepatocellular carcinoma formation. Proteasomal activity seems to be an important factor in cancerogenesis, since proteasomal activity is altered in many cancer types (Table 3) and the use of proteasome activators and inhibitors as anti-cancer therapeutics showed promising results [100, 164, 165]. In most cancer types, proteasomal activity is elevated [75–89]. Until now, the reason for this increase is unknown. Since mutations of genes encoding ciliary proteins led to a reduced proteasomal activity in ciliopathies of mice and zebrafishes [122, 126], it might seem as if mutations in these genes could only play a role in cancer types with reduced proteasomal activity. However, it was reported that RPGRIP1L controls the ciliary proteasome in MDCK cells negatively opposing the findings in MEFs and embryonic mouse limbs [126, 166]. These findings as well as studies on cilia length argue for a cell type-specific function of RPGRIP1L allowing that mutations in *RPGRIP1L* cause an increase of ciliary proteasome activity in some organs and a concomitant reduction of this activity in other organs [126]. Theoretically, it is conceivable that an increased amount of ciliary proteins leads to enhanced proteasomal activity. In this regard, a recent study demonstrated that the overexpression of the RPGRIP1L domain, which interacts with the proteasomal component PSMD2, gives rise to an elevated activity of the ciliary proteasome [126]. What remains to be determined is if the increased proteasomal activity found in most cancer types could be due to impaired regulation of proteasomal activity by ciliary proteins.

**Table 2:** Ciliary presence in different cancer types

Cancer type	Cancer cell type	Ciliary presence		Cilia-associated information	References
		Yes	No		
Bladder cancer	Urothelial cells; urothelial carcinoma (UC) cell lines	X		Cilia-associated HH signaling mediates the proliferation and survival of human urothelial carcinoma (UC) cell lines and is required for UC tumor growth in vivo	[58]
Brain tumor	Medulloblastomas	X	X*	Anaplastic* medulloblastomas have few or no ciliated cells; cilia are present in most desmoplastic medullablastoma but almost exclusively in tumors that have activation in either HH or WNT signaling	[59]
Breast cancer	Breast cancer cells; breast cancer cell lines	X**	X	Absence of primary cilia; loss of primary cilia in all non-proliferating human tumor cells; (decreased in amount)**	[151, 152**, 153]
Cancer stem cells	Medulloblastoma stem cells		X	CD15 + medulloblastoma cells lack primary cilia	[150]
Colon cancer	Colon epithelium cells	X		Decreased frequency of primary cilia in absence of TTLL3 linked to the development of human colorectal carcinomas	[154]
Lung cancer	Columnar epithelium; mucous columnar cells	X	X***	Loss of cilia by change from normal ciliated columnar epithelium to mucous columnar cell in cases of non-terminal respiratory unit type adenocarcinoma***	[155]
Skin cancer	Melanocytes	X****	X	Decreased amount of primary cilia in melanocytes****; loss of primary cilia in melanoma cell lines	[156]
Ovarian cancer	Epithelial ovarian cells	X		Reduced cilia frequency; deregulated Hh and platelet-derived growth factor receptor alpha (PDGFRα) signaling	[57]
Pancreatic cancer	Pancreatic ductal cells; pancreatic cancer cell lines	X	X	Primary cilia were identified in pancreatic cancer cell lines and in 25 of 100 pancreatic ductal adenocarcinoma (PDAC) cases; the presence of primary cilia is significantly associated with the prognosis of PDAC	[157]
Prostate cancer	Prostatic epithelial cells	X		Reduced primary cilia frequency; tendency to shorter cilia	[158]
Renal cancer	Renal cells; renal tumor parenchyma	X		Strongly reduced cilia frequency; the reduction in clear cell renal cell carcinomas (ccRCC) is significantly stronger than in papillary renal cell carcinomas (pRCC)	[159–161]

**Table 3:** Status of proteasomal activity in different cancer types

Cancer type	Cancer cell type	Status of proteasomal activity		References
		Increased proteasomal activity	Reduced proteasomal activity	
Bladder cancer	Human bladder cancer cells; human T24 urinary bladder carcinoma cell line	X		[75–77]
Brain tumor	GBM stem-like cells; temozolomide-resistant glioma cell lines	X		[104]
Breast cancer	MCF-7 and MDA-MB-231 human breast cancer cells	X		[78]
Cancer stem cells	Various cancer stem cells; human head and neck squamous cell carcinoma (HNSCC) cells; breast cancer stem cells (BCSCs)		X	[98–103]
Colon cancer	HCT116 colon adenocarcinoma cells; metastatic colorectal cancer tissue	X		[79, 80]
Lung cancer	Lung cancer cell lines (H460, A549 and H129)	X		[81, 82]
Skin cancer	Various human and mouse tumor cell lines	X		[84]
Ovarian cancer	Various ovarian cancer cell lines	X		[83]
Pancreatic cancer	MIA-PaCa-2 human pancreatic cancer cells	X		[85]
Prostate cancer	LNCaP (AD) and PC3 (AI) PCa cells	X		[86]
Renal cancer	Renal cell carcinoma tissue; clear cell renal cell carcinoma (CCRCC) cell lines	X		[87–89]

Another cancer cell type in which the cilia-regulated proteasome might play a leading role is the CSC. Since the loss of ciliary proteins BBS4, BBS7, OFD1, and RPGRIP1L resulted in a reduced proteasomal activity [50, 122, 123, 126] and CSCs lack cilia in addition to a decreased proteasomal activity [98–103, 150], it is quite possible that a reduction of cilia-regulated proteasomal activity causes the development and/or ensures the survival of most CSCs. However, this is more of a meta-analysis. The only kind of CSC reported to lack cilia was a medulloblastoma CSC [150]. Until now, data about the existence of cilia on other CSCs are missing. Consequently, the presence of cilia in CSCs of other cancer types needs to be investigated. To gain insight into the potential relationship between the cilia-regulated proteasome and cancerogenesis, it is necessary to perform comparative investigations focusing on the activity of the ciliary proteasome and the presence of cilia in cancer cells.

## Conclusion

Oncogenic signaling pathways are mediated by primary cilia. Consequently, an association between primary cilia and cancer is very likely. Altered proteasomal activity is an often observed feature in cancer cells [75–89, 98–103] and it was demonstrated that ciliary proteins control proteasomal activity [50, 122, 123, 126]. Previously, it was suggested that the dysfunction of the cilia-controlled proteasome is only one contributory factor of the ciliopathic pathology [122]. Thus, an important purpose of future studies will be to reveal the impact of the cilia-regulated proteasome in human ciliopathies. This aim is closely related to the analysis of the cilia-regulated proteasomal activity in cancer. Consequently, cancer therapies could be advanced by targeting cilia. In the context of proteasomal activity, SFN is a promising therapeutic agent for ciliopathies and any form of cancer in which proteasomal activity is reduced. It remains an open question whether the reduced activity in these cancer types corresponds to the cilia-controlled proteasomal activity. The answer to this question could extend the knowledge about oncogenic factors in a significant direction. Interestingly, a characteristic of most CSCs is a decreased proteasomal activity [98–103] making it possible that new insights into the field of cilia and in particular, the cilia-regulated proteasome, help to understand the biology of tumor formation and reformation as well as the therapeutic possibilities to treat various types of cancer. However, even if nearly all CSCs display a reduced proteasomal activity, most cancer types exhibit the exact opposite—an elevated proteasomal activity. There is scant evidence of ciliary dysfunction resulting in an increase of proteasomal activity, but it does not seem to be impossible due to cell type-specific functions of ciliary proteins [126, 166]. In this regard, it would be helpful to know whether the higher proteasomal activity in cancer cells depends on “proteotoxic crisis” or not [91].

Based on the novelty of the relationship between the primary cilium and the proteasome, it is difficult to make a clear statement to the role of the cilia-regulated proteasome in cancerogenesis. However, this research topic is very promising and the relationship between the cilia-controlled proteasome and cancer holds enormous potential for the development of new anti-cancer therapies.

## Abbreviations

AKT/PKB: protein kinase B
ALS: Alström syndrome
APC: adenomatous polyposis coli
BB: basal body
BBS: Bardet–Biedl syndrome
CCCI: cilia/centrosome complex interactome
CDC37: cell division cycle control protein 37
CED: sensenbrenner syndrome (cranioectodermal dysplasia)
CK1: casein kinase 1
CSC: cancer stem cell
DNA: deoxyribonucleic acid
DSH: disheveled
ERK: extracellular signal-regulated kinases
EVC: Ellis–van Creveld syndrome
FGF: fibroblast growth factor
FTM: fantom
FZ: frizzled
GLI: glioblastoma
GLI2/3-A: glioblastoma 2/3 activator
GLI2/3-R: glioblastoma 2/3 repressor
LAP: localization and affinity purification
GSC: glioma stem-like cell
GSK3: glycogen synthase kinase 3
HSP90: heat shock protein 90
INVS: inversin
IQCB1: IQ motif containing B1
JATD: Jeune asphyxiating thoracic dystrophy
JBTS: Joubert syndrome
JNK: C-Jun N-terminal kinases
LCA: Leber’s congenital amaurosis
LRP 5/6: low density lipoprotein-related proteins 5/6
MAD2: mitotic arrest deficient 2
MDCK: Madin-Darby canine kidney cell line
MEF: mouse embryonic fibroblasts
MEK: mitogen-activated protein kinase
MKS: Meckel–Gruber syndrome
mRNA: messenger ribonucleic acid
NIC: NOTCH intracellular domain
NPHP: nephronophthisis
OFD1: orofaciodigital syndrome type 1
PCP: planar cell polarity
PDGF: platelet-derived growth factor
PDGFRα: platelet-derived growth factor receptor-α
PSMA5: proteasome subunit alpha type-5
PSMD2: proteasome 26S subunit, non-ATPase, 2
PSMD3: proteasome 26S subunit, non-ATPase, 3
PSMD4: proteasome 26S subunit, non-ATPase, 4
PTCH1: patched1
RBP-J/CBF1/CSL: recombining binding protein suppressor of hairless
RHO A: ras homolog gene family, member A
ROCK: rho-associated protein kinase
RPGRIP1L: retinitis pigmentosa GTPase regulator-interacting protein-1 like
SFN: sulforaphane
SHH: sonic hedgehog
SLS: Senior–Løken syndrome
SMAD: SMA- and MAD-related proteins
SMO: smoothened
TGFβ: transforming growth factor-β
TGFβ-RI/II: transforming growth factor β receptor I/II
VANGL2: van gogh-like 2
WNT: wingless/integrated
TZ: transition zone
